# Clinical and histopathological study of a hollow and posteriorly multiperforated polymethylmethacrylate implant in eviscerated rabbit eyes

**DOI:** 10.5935/0004-2749.20230064

**Published:** 2023

**Authors:** Marlos R Lopes e Silva, Fernando Chahud, Antonio Augusto V. Cruz

**Affiliations:** 1 Department of Ophthalmology, Otorhinolarymgology and Head and Neck Surgery, Hospital das Clínicas, Faculdade de Medicina de Ribeirão Preto, Universidade de São Paulo, Ribeirão Preto, SP, Brazil.; 2 Department of Pathology, Hospital das Clínicas, Faculdade de Medicina de Ribeirão Preto, Universidade de São Paulo, Ribeirão Preto, SP, Brazil.

**Keywords:** Orbital implants, Polymethylmethacrylate, Eye evisceration, Anophthalmos, Ophthalmological surgical procedures, Rabbits, Implantes orbitários, Polimetilmetacrilato, Evis­ceração ocular, Anoftalmia, Procedimentos cirúrgicos oftal­mológicos, Coelhos

## Abstract

**Purpose:**

The study aimed to evaluate the clinical and tissue response to a hollow polymethylmethacrylate orbital implant with a multiperforated posterior surface in an animal model after evisceration.

**Methods:**

Sixteen New Zealand rabbits had their right eye eviscerated. All animals received a hollow polymethylmethacrylate implant 12 mm in diameter that is multiperforated in its posterior hemisphere. The animals were divided into four groups, and each one had the eye exenterated at 7, 30, 90, and 180 days post-evisceration. Clinical signs were assessed daily for 14 days post-evisceration and then every 7 days until 180 days. Inflammatory pattern, collagen structure, and degree of neovascularization generated with implant placement were analyzed with hematoxylin-eosin, picrosirius red, and immunohistochemistry staining.

**Results:**

There were no signs of infection, conjunctival or scleral thinning, or implant exposure or extrusion in any animal during the study. On day 7, the new tissue migrated into the implant and formed a fibrovascular network through the posterior channels. Inflammatory response reduced over time, and no multinuclea­ted giant cells were found at any time.

**Conclusion:**

Hollow polymethylmethacrylate orbital implants with a multiperforated posterior surface enable rapid integration with orbital tissues by fibrovascular ingrowth. We believe that this orbital implant model can be used in research on humans.

## Abstract (pt)

**Objetivo:**

Avaliar a resposta tecidual e clínica a um implante orbitário de polimetilmetacrilato, oco e multiperfu­rado em sua porção posterior em modelo animal após evisceração.

**Métodos:**

Dezesseis coelhos da raça Nova Zelândia foram submetidos à evisceração do globo ocular direito. Todos receberam implante oco de polimetilmetacrilato de 12 mm de diâmetro, multiperfurado em sua semiesfera posterior. O estudo foi dividido em avaliação clínica e histopatológica. A avaliação clínica foi diária até 14 dias pós-evisceração e, a cada sete dias, até completar 180 dias. Os animais foram divididos em grupos de quatro animais e cada um foi submetido à exenteração com 07, 30, 90 e 180 dias e depois à eutanásia. A análise histopatológica teve por fim caracterizar o padrão inflamatório, a estrutura do colágeno e o grau de neovascularização. Para isso, além da tradicional coloração pela hematoxilina-eosina, utilizou-se o corante Picrosirius Red (PSR) e imuno-histoquímica com o marcador CD 34.

**Resultados:**

Não houve sinais de infecção, afinamento conjuntival ou escleral, exposição ou extrusão do implante em nenhum animal durante o estudo. Já no sétimo dia, o tecido neoformado migrou para dentro do implante formando uma rede fibrovascular através dos canais posteriores. A resposta inflamatória diminuiu ao longo do tempo avaliado e não foram encontradas células gigantes multinucleadas.

**Conclusão:**

O implante analisado permite a sua integração aos tecidos orbitários pelo crescimento fibrovascular em seu interior. Os autores acreditam que este modelo de implante orbital pode fazer parte de testes com humanos.

## INTRODUCTION

Orbital implants are crucial for volume reconstruction of anophthalmic sockets and allow for proper adaptation of an ocular prosthesis. Implants can be classified as integrated or nonintegrated. Integrated implants favor fibrovascular tissue ingrowth and penetration, leading to implant integration into the socket^(1)^. This biocompatibility depends not only on the material porosity but also on the composition variability, mechanical features, and material microstructure^(2)^.

Few studies have analyzed the surface of polymethylmethacrylate (PMMA) implants; however, this inert material is characterized by a minimal inflammatory response to tissues, decreased roughness among biomaterials options, and potential use in different implant designs^(3)^. PMMA implants were previously considered nonintegrated, but different designs of perforated implants enabled them to support fibrovascular ingrowth, and therefore, integration with orbital tissues^(4,5)^.

The evolution of integrated implants in nonporous materials occurred in solid models and began in 1940 with Allen, whose implant had a peg connected to a prosthesis. This implant inspired Iowa in 1959 to develop a new model without a peg, with depressions that favored the fit and interconnection between muscles and holes that allowed tissue penetration. In 1987, Universal implant appeared as a variation of Iowa implant, with deeper depressions and more rounded mounds; however, its use was suppressed by the new generation of porous materials^(4)^.

The use of a hollow model was revolutionary in orbit reconstruction when P.H. Mules, in 1885, described the evisceration technique using a spherical hollow glass implant. The fragility of the material to impact and sudden temperature changes made its use unfeasible^(4)^. In 2004, a comparative study between hollow and solid PMMA spheres with analysis of density, strength, and water absorption identified hollow spheres as a substitute for solid spheres, despite the limitations of the study^(6)^. To date, the use of a hollow, multiperforated sphere in anophthalmic sockets has not been described in the literature.

In 2013, a study on a multiperforated PMMA implant model found tissue ingrowth into the sphere, even though the material was nonporous^(7)^. They reported that the implant integrated with the tissues without migration, extrusion, or sphere exposure^(7)^. In that study, they observed that the sclera became thinner at the anterior surface of the sphere^(7)^. One option to avoid scleral thinning was to use barriers that protect the conjunctival surface from the porosity of integrated implants^(8)^. Although they reduce the risks of exposure and extrusion, these materials increase the cost and time of surgery^(9,10)^. We proposed a new implant model in which the perforations are restricted to the posterior surface to avoid scleral thinning and/or the use of barriers covering the implant.

This improved model was an object of analysis in this study. In an animal model, we evaluated clinical and tissue response to a hollow PMMA orbital implant with a multiperforated posterior surface and unperforated anterior surface.

## METHODS

The experiment followed the ethical principles of animal experimentation adopted by Brazilian College of Animal Experimentation (COBEA) and the ethical considerations in biomedical research provided by Association for Research in Vision and Ophthalmology. It was approved by Animal Care and Use Committee of the School of Medicine of Ribeirão Preto of University of São Paulo, Brazil, on September 24, 2012.

**Figure 1:**
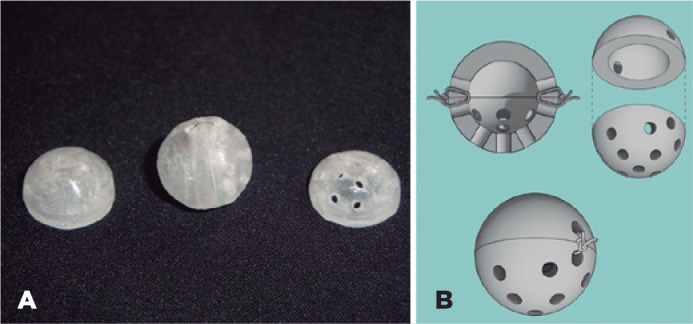
Hollow polymethylmethacrylate orbit implant, formed by the union of two hemispheres. A) Actual appearance of the implant. B) Hollow implant prototype.

The implant was composed of two hollow hemispheres of PMMA, 12 mm in diameter and 2 mm in wall thickness, manufactured using matrices, and developed in Laboratory of the Department of Dental Materials and Prosthetics of the School of Dentistry of Ribeirão Preto, University of São Paulo. In the posterior hemisphere, 13 circular holes of 1.5-mm diameter were made manually with a perforating drill. The regularities and angulations of the holes for the formation of channels were of the same number and standardized. The anterior hemisphere had only two holes on the sides to allow the passage of a 6-0 nylon thread and fixation with the posterior hemisphere (Figure 1). The hemispheres were polished and sent for sterilization by ethylene oxide, under the responsibility of the company *Oximed Tecnologia em Esterilização Ltda.*

Sixteen male New Zealand rabbits weighing on average 3,000 g had their right eye eviscerated. The animals were initially sedated with 0.2% acepromazine (1 mg/kg of body weight) administered intramuscularly (IM), and then anesthetized 10 minutes later with ketamine hydrochloride (30 mg/kg IM). One drop of topical proxymetacaine and a peribulbar injection of 1 ml of 2% lidocaine hydrochloride were also instilled and performed, respectively.

The right eye of each animal was eviscerated with 360-degree posterior sclerotomy using a number 11 scalpel blade. The orbital implant was inserted with the caution of keeping the multiperforated side posteriorly into the socket. The anterior opening of the sclera was sutured with 4-0 silk, and the Tenon’s capsule and conjunctiva were sutured separately with 6-0 vicryl. As a preventive measure against infection, a drop of 0.5% moxifloxacin (Vigamox^®^, Alcon, Brazil) was instilled every 12 hours for 3 consecutive days. After 3 days, there was no cavity cleaning or eye drops use.

The rabbits were divided into groups according to the day they were euthanized (7, 30, 90, and 180 days postoperatively [PO]). The following clinical signs were recorded: chemosis, ocular discharge, hemorrhage, suture dehiscence, and implant exposure and extrusion. The 7-day PO group was clinically assessed daily for 7 days. The other groups were assessed daily for 14 days and then every 7 days until the day of exenteration. Clinical signs, such as chemosis, hemorrhage, and ocular secretion, were quantified in crosses, with (-) for absent, (+) for mild, (++) for moderate, and (+++) for intense. For suture dehiscence, implant exposure and extrusion were considered (-) when absent and (+) when present.

For exenteration, the rabbits were sedated with 0.2% acepromazine (1 mg/kg of body weight IM), and then anesthetized 10 minutes later with ketamine hydrochloride (30 mg/kg) and xylazine hydrochloride (5 mg/kg). A drop of topical proxymetacaine was instilled into the right eye. Sodium thiopental (20 mg/kg) was administered intravenously to maintain analgesia during surgery and as an overdose (40 mg/kg) to euthanize the animals. The surgery consisted of a periorbicular incision to remove all orbital contents, including the implant.

The exenterated orbital contents were washed with 0.9% saline and immersed in 10% buffered formalin for 48 hours. The implants were then carefully separated from the orbital tissues, and any newly formed tissue was embedded in paraffin. Hematoxylin-eosin (HE), picrosirius red (PSR), and CD34 staining were used to characterize tissue response to the implants.

Three photographs of each of the 16 HE-stained slides were taken at an 400× magnification for quantitative microscopic analysis of the inflammatory cells. In a qualitative analysis of the connective tissue, one photograph was taken for each of the 16 PSR-stained slides under a bright backlight at a 100x magnification. For the counting of capillary loops, three photographs of each of the 16 slides were taken, and an immunohistochemical study was performed with CD34 marker. The photographed areas were selected by a pathologist who, after evaluating the unidentified slides, chose “hot spots,” which contained the highest concentrations of inflammatory cells or connective tissue.

In each photograph, the number of blood vessels, inflammatory cells, and polymorphonuclear (PMN) and mononuclear neutrophils was manually counted with the help of Image J software, version 1.42. Immunohistochemistry with CD34 marker was used to facilitate the identification of new vessels. The number of vessels and inflammatory cells was added up based on three photographs of each slide, with the mean and median being used in event analysis in addition to the Kruskal-Wallis nonparametric analysis of variance.

Collagen formation and maturation were analyzed qualitatively by the histochemical method of PSR staining. On microscopy with this method, collagen fibers appear red and, under polarized light by birefringence, type I collagen appears as thick yellow-orange fibers. Type III collagen, in turn, appears as thinner greenish, less compacted fibers. Collagen birefringence correlates with collagen brightness when viewed under polarized light.

## RESULTS

**Figure 2:**
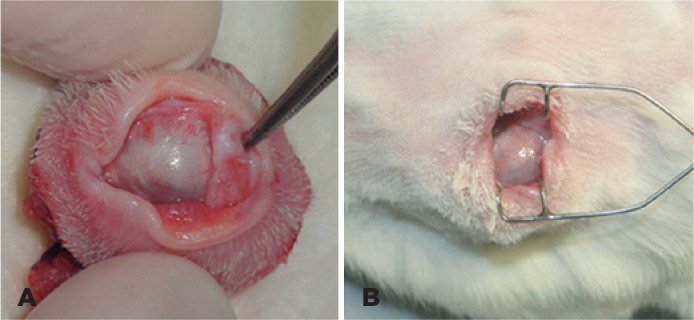
Appearance of the cavity after 90 days (A) and 180 days (B) of evisceration.

Mild (+) to moderate (++) presence of mucoid secretion in the orbital cavity was observed in all animals on the first day PO, decreasing to absent (-) during the study period. Chemosis was present in mild (+) to moderate (++) form on the first days PO, evolving to absent (-) during the study period. There was no hemorrhage, suture dehiscence, conjunctival or scleral thinning, or implant exposure or extrusion in any animal (Figure 2).

**Figure 3:**
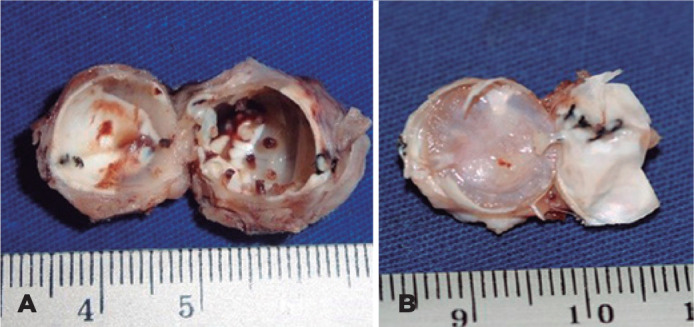
Projections observed inside the implant after 7 days of evisceration (A) and exenterated orbital implant 180 days post-evisceration (B).

Tissue colonization was observed within the implant canals from day 7 PO, together with the formation of a pseudocapsule surrounding the sclera (Figure 3A). In the last study group, 180 days PO, dense newly formed tissue was noted in almost the entire interior of the implant (Figure 3B). The separation of the newly formed tissue in the perforated implant portion was difficult because of dense and fibrotic tissue inside the implant and the thick pseudocapsule over the sclera.

**Figure 4:**
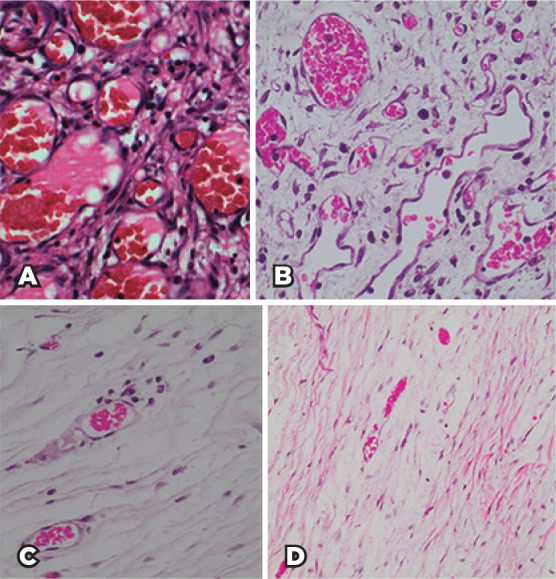
Pattern of distribution of inflammatory cells according to postoperative time points (A: 7 days; B: 30 days; C: 90 days; D: 180 days). There is great initial inflammatory activity inversely proportional to the formation of collagen fibers (hematoxylin-eosin staining, 400×).

Histologic analysis noted an inflammatory infiltrate consisting of PMN neutrophils, lymphocytes, and macrophages at 7 days PO. This infiltrate progressively reduced in 30- and 90-day PO groups. Few inflammatory cells were found in 180-day PO group (Figure 4 and Table 1). No multinucleated giant cells or epithelioid cells were found in the inflammatory infiltrate.

**Table 1:** Number of inflammatory cells and blood vessels per animal

Group	Animal	Inflammatory cells (N)		Blood vessels (N)	
		Median	Mean	Median	Mean
7 days	1	24.0	26.7	7.0	7.3
	2	26.0	20.7	14.0	12.0
	3	13.0	12.7	6.0	5.7
	4	23.0	24.7	11.0	12.3
30 days	13	2.0	2.0	4.0	4.0
	14	3.0	2.7	4.0	3.7
	15	5.0	5.3	7.0	7.7
	16	4.0	3.7	6.0	7.3
90 days	9	3.0	2.7	4.0	4.3
	10	1.0	1.0	3.0	2.7
	11	1.0	1.0	3.0	3.7
	12	1.0	1.3	4.0	3.7
180 days	5	1.0	1.3	3.0	2.7
	6	1.0	0.7	2.0	1.7
	7	1.0	1.0	3.0	3.7
	8	0.0	0.3	3.0	3.0

The values listed represent the parameters of three measurements per animal.

**Figure 5:**
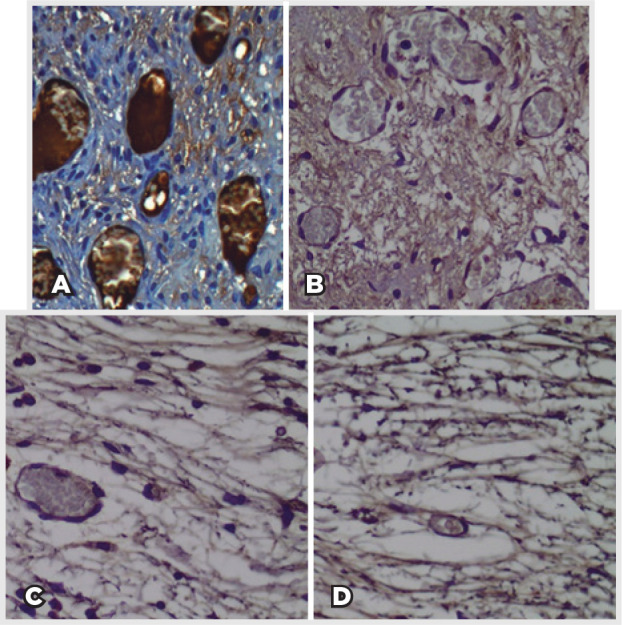
New blood vessels marked with CD34 in tissue ingrowths according to postoperative time points (A: 7 days; B: 30 days; C: 90 days; D: 180 days). The minimum number of new vessels increased over time.

New vessel quantification with immunohistochemical marker showed greater angiogenic activity in 7-day PO group, with a progressive reduction in 30- and 90-day PO groups. A small number of new vessels were observed in 180-day PO group, and they had matured endothelial cells remodeling the capillaries (Figure 5 and Table 1).

**Figure 6:**
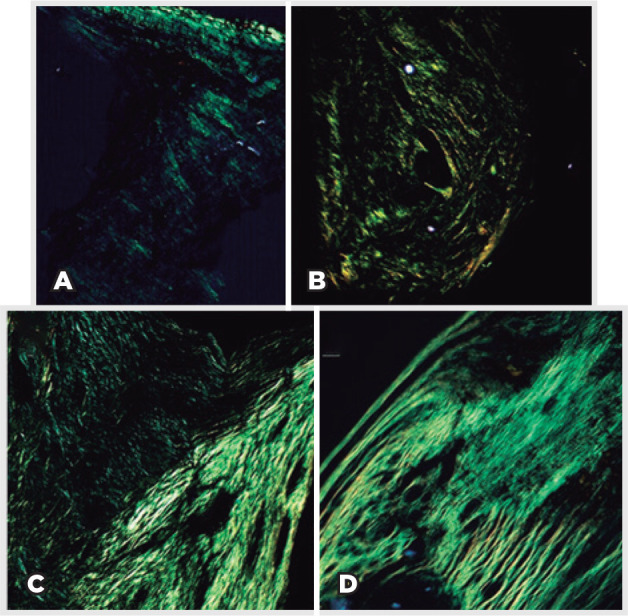
Collagen fibers in tissue ingrowths according to postoperative time points (A: 7 days; B: 30 days; C: 90 days; D: 180 days) under polarized light (picrosirius red staining, 100×).

Connective tissue showed an increase in fibroblasts and transformation of type III collagen into type I collagen, which has greater tensile strength. PSR staining under polarized light revealed thin and loose collagen fibers (stained green) at 7 days PO. At 90 days PO, collagen fibers became denser, as shown by the predominantly yellow stains under polarized light. Finally, at 180 days PO, maturation of collagen fibers was achieved with greater density and tensile strength, as demonstrated by the yellow stains under polarized light (Figure 6).

## DISCUSSION

The study on new implant models for anophthalmic socket reconstruction aimed to improve tissue integration from the implant to the host and reduce the biological response to the implanted material. In our study, the hollow PMMA implant model with a multiperforated posterior surface showed good biocompatibility, low inflammatory reaction, and tissue integration similar to that observed in integrated implants. Fibrovascular tissue penetration was related to holes and not to the material porosity. No animals had migration, exposure, or extrusion of the implant.

This implant was easy to sterilize, as the structure of two hollow hemispheres favors practicality and safety in the cleaning and sterilization process. Also, it was inexpensive and easy to prepare.

The study model allows integration because fibrovascular tissue penetrates through the channels in the posterior surface of the hollow implant, demonstrating physical integration. Tissue ingrowth into the perforations was seen in animals exenterated at 7 days PO, which is explained by the larger diameter of the holes and by 360-degree posterior sclerotomy, allowing the use of even larger implants^(11)^. This pattern of tissue ingrowth inside the implants was similar to those in studies on integrated porous polyethylene implants^(12,13)^. Because of the implant unperforated anterior surface, no scleral thinning was found during the study period; this distinguishes our implant from other implants whose porous material or perforated model favors scleral thinning. Another advantage was its low weight, resulting from being a hollow sphere. Lightweight implants reduce the possibility of migration and extrusion due to a lower action of gravity on weight^(6,14)^.

Low inflammatory activity with mononuclear and PMN cells was observed at all-time points, with a trend toward reduction throughout the study. The more intense inflammatory reaction seen at baseline was considered a tissue response to the induced surgical trauma and not a reaction to the implant. Another important finding was the absence of multinucleated giant cells in the tissues, which rules out a foreign-body granulomatous inflammatory reaction to the implant. Multinuclea­ted giant cells have been seen in integrated implants (hydroxyapatite and porous polyethylene) but with low inflammation and no clinical manifestations^(13)^. The authors suggested that the absence of granulomatous inflammation was due to the superficial regularity of the implant, a situation already observed by Choi et al. in a comparative histological study between PMMA, porous polyethylene, and hydroxyapatite implants^(3)^.

In addition to the absence of giant cell reaction, implant integration was observed by the process of collagen fibers maturation, as shown by PSR staining under polarized light. The replacement of hyaluronic acid and fibronectin deposits with types I and III collagen was similar to that observed in the physiological healing. Type III collagen (stained green) is the collagen of granulation tissue produced by young fibroblasts. Then, type I collagen is formed as the final product of tissue healing. The deposition of these two collagen types promotes scar tissue formation with greater tensile strength^(15)^.

Angiogenic activity assessed with CD34 immunohistochemistry was similar to that observed in the physiological healing process. There was intense tissue neovascularization at 7 days PO and little angiogenic activity at 180 days PO with mature endothelial cells remodeling the capillaries. PMMA does not have angiogenic growth factors on its surface that lead to stimulated vascularization, as is the case with 45S5 Bioglass^®(16)^.

Recent studies have extended the concept of tissue integration to implants and considered the response of material surface chemical composition to biological fluids. These studies showed that ion exchange mechanisms, microstructure, and mechanical features are key factors for adequate fibrovascularization and consequent success in tissue integration to the implant^(2,17)^. This new knowledge about materials bioactivity adds to the importance of interconnection, number, and size of pores as a factor for tissue integration to the implant^(1,17)^.

To date, few studies have analyzed the surface of a PMMA implant. Still, its decreased roughness among biomaterial options, minimal inflammatory response to tissues, and the formation of a pseudocapsule around it are known^(3)^. However, despite being a nonporous material, it allows the physical integration of tissue depending on the implant model used^(18)^.

Based on the results obtained in this study, we concluded that the hollow PMMA implant model with a multiperforated posterior surface and unperforated anterior surface can be a new implant option that enables rapid integration with orbital tissues by fibrovascular ingrowth. Also, we believe it can be evaluated in a phase 3 study.
